# Monoclonal Antibodies From Anti-NMDA Receptor Encephalitis Patient as a Tool to Study Autoimmune Seizures

**DOI:** 10.3389/fnins.2021.710650

**Published:** 2021-08-26

**Authors:** Olga Taraschenko, Howard S. Fox, Ember Eldridge, Wenyi Wang, Samuel W. Dowd, Fetweh Al-Saleem, Chandana Devi Kattala, Scott K. Dessain, Raymond Dingledine

**Affiliations:** ^1^Department of Neurological Sciences, Division of Epilepsy, University of Nebraska Medical Center, Omaha, NE, United States; ^2^Department of Pharmacology and Chemical Biology, Emory University School of Medicine, Atlanta, GA, United States; ^3^Lankenau Institute for Medical Research, Wynnewood, PA, United States

**Keywords:** anti-NMDA receptor encephalitis, monoclonal antibodies, autoimmune seizures, cytokines, neuronal autoantibodies

## Abstract

Anti-N-methyl-D-aspartate (NMDA) receptor encephalitis manifests with precipitous cognitive decline, abnormal movements, and severe seizures that can be challenging to control with conventional anti-seizure medications. We previously demonstrated that intracerebroventricular (i.c.v.) administration of cerebrospinal fluid from affected patients, or purified NMDA receptor antibodies from encephalitis patients to mice precipitated seizures, thereby confirming that antibodies are directly pathogenic for seizures. Although different repertoires of anti-NMDA receptor antibodies could contribute to the distinct clinical manifestations in encephalitis patients, the role of specific antibodies in the expression of seizure, motor, and cognitive phenotypes remains unclear. Using three different patient-derived monoclonal antibodies with distinct epitopes within the N-terminal domain (NTD) of the NMDA receptor, we characterized the seizure burden, motor activity and anxiety-related behavior in mice. We found that continuous administration of 5F5, 2G6 or 3C11 antibodies for 2 weeks precipitated seizures, as measured with continuous EEG using cortical screw electrodes. The seizure burden was comparable in all three antibody-treated groups. The seizures were accompanied by increased hippocampal C-C chemokine ligand 2 (CCL2) mRNA expression 3 days after antibody infusion had stopped. Antibodies did not affect the motor performance or anxiety scores in mice. These findings suggest that neuronal antibodies targeting different epitopes within the NMDA receptor may result in a similar seizure phenotype.

## Introduction

Anti-N-methyl-D-aspartate receptor (NMDAR) encephalitis is a devastating autoimmune disease that manifests with precipitous cognitive decline, hallucinations, and seizures in previously healthy persons with mortality approaching 7–8% (Dalmau et al., 2008). Controlling the seizures and psychiatric symptoms in the acute period constitutes the main clinical challenge, while managing cognitive deficits in the chronic phase of encephalitis is the primary goal of rehabilitation. Previous studies in rodents confirmed that both cognitive deficits and seizures are precipitated by direct pathogenic effects of antibodies (Planaguma et al., 2015; Taraschenko et al., 2019). Produced by the intrathecal and peripherally circulating memory B cells, antibodies are formed against the corresponding receptor targets in the central nervous system (CNS) or in peripherally located tumors leading to the manifestations of primary autoimmune or paraneoplastic encephalitis, respectively (Dalmau, 2016; Makuch et al., 2018). The disruption of the blood brain barrier that occurs in anti-NMDAR encephalitis or infections facilitates the entry of antibodies and B-cells into the cerebrospinal fluid (CSF), that is thought to prime the acute manifestations of encephalitis (Hammer et al., 2014; Castillo-Gomez et al., 2016; Omae et al., 2018). Observations that reduction in the CSF antibody titers in patients correlates with an improvement of encephalitis symptoms further supports the premise of antibody pathogenicity (Gresa-Arribas et al., 2014); however, the specific pathophysiologic mechanisms associated with variable clinical phenotypes of the autoimmune encephalitis remain unclear.

On the cellular level, patient polyclonal anti-NMDAR antibodies bind to epitopes located at the amino terminal domain (ATD) of the GluN1, cause cross-linking and internalization of the surface NMDAR clusters, and the reduction of postsynaptic currents (Hughes et al., 2010; Gleichman et al., 2012; Mikasova et al., 2012; Warikoo et al., 2018). The incubation of anti-NMDAR antibodies with brain tissue *in vitro* results in reduced synaptic plasticity and profound alteration of long-term potentiation (LTP) (Mikasova et al., 2012). The morphological and electrophysiological changes caused by patient CSF do not depend on the involvement of any other neuronal antibody subtypes or complement activation, suggesting that anti-NMDAR antibodies alone are sufficient for encephalitis pathogenesis (Kreye et al., 2016). When infused or injected into the rodent brain, human polyclonal anti-NMDAR antibodies bind primarily in the hippocampus and are associated with impaired novel object recognition and prepulse inhibition behavior as well as spontaneous seizures and a reduction of the seizure threshold to chemical proconvulsants (Planaguma et al., 2015; Wright et al., 2015; Taraschenko et al., 2019; Carceles-Cordon et al., 2020). Similar to the effects of polyclonal anti-NMDAR antibodies found in patient CSF, monoclonal anti-NMDAR antibody recovered from an encephalitis patient demonstrated epitope specificity and key cellular effects similar to those of polyclonal antibodies (Malviya et al., 2017). Further, the administration of the patient’s monoclonal antibodies to mice resulted in a diminished novel object recognition function and impaired motor phenotype (Malviya et al., 2017; Sharma et al., 2018a).

The effects of the depletion of NMDARs on behavior in patients and rodent models resemble the phenotypic effects of pharmacological blockade or genetically induced reduction of NMDAR function, including profound cognitive impairment, psychosis, and seizures (Davis et al., 1992; Tsien et al., 1996; Jentsch and Roth, 1999; Bey and Patel, 2007). The profile of symptoms caused by antagonists of the NMDAR may rely on the dose of antagonist and the resulting functional and structural alterations of NMDAR-related synaptic networks (Krystal et al., 1994; Bey and Patel, 2007; Masdeu et al., 2016). The spectrum of clinical manifestations may also depend on the repertoire of various monoclonal anti-NMDAR antibodies that can co-exist in the same patient and have different epitope profiles or downstream effects (Kreye et al., 2016). Using the mouse model of autoimmune seizures in encephalitis developed in our laboratory, we previously studied the epileptogenic effects of a monoclonal antibody isolated from a patient with anti-NMDA receptor encephalitis (Taraschenko et al., 2019, 2021). We found that the antibodies induce seizures that can be inhibited by anakinra, a selective IL-1 receptor antagonist. Here, we extend these studies, including the addition of two other antibodies from the same patient, to determine the effects of prolonged exposure on key phenotypic characteristics of anti-NMDAR encephalitis, including anxiety, seizure, and motor phenotype. In addition, we examine the effects of antibody-induced seizures on the expression of relevant inflammatory mediators in the hippocampus of mice. We previously showed that the antibodies bind at distinct sites on the amino-terminal domain of GluN1, but both require the presence of N368 for binding (Sharma et al., 2018a). Upon binding the NMDA receptors in hippocampal neurons, they internalize via a mechanism that is inhibited by the NMDA receptor channel blocker, MK-801 (Sharma et al., 2018a).

## Materials and Methods

### Animals

All experiments were conducted in accordance with the National Institutes of Health Guide for the Care and Use of Laboratory Animals and were approved by the Institutional Animal Care and Use Committee (IACUC) of the University of Nebraska Medical Center (UNMC). The principles outlined in the ARRIVE 2.0 guidelines (Percie du Sert et al., 2020) and the Basel declaration, including the 3R concept, were followed during experimental planning.

Male C57BL/6 mice (8–10 weeks, 25–30 g from Charles River, Roanoke, IL) were housed in groups of five and maintained on a 12 h light cycle (light on/off at 7 a.m./7 p.m.) with *ad libitum* access to food and water. Following the implantation of the intracerebroventricular (i.c.v.) guide cannula, EEG electrodes and head mount, mice were placed in the recording chambers individually until the completion of EEG monitoring.

### Hybridoma Generation and Antibody Purification

IgG1λ monoclonal antibodies (5F5, 2G6) specific for GluN1 were derived from memory B cells of a patient with anti-NMDAR encephalitis and seizures as previously described (Sharma et al., 2018a). Specific binding of the NMDA receptor was confirmed using indirect immunofluorescence on primary hippocampal neurons and a 293T cell line that ectopically expresses the GluN1 ATD (Sharma et al., 2018b). The 3C11 IgG was cloned from the same patient using On-Cell monoclonal antibody (mAb) Screening (Puligedda et al., 2019). The 3C11 antibody also binds to the hippocampal GluN1 in the ATD (Puligedda et al., 2019). The 6A IgG1λ human monoclonal antibody does not bind GluN1 and was used as an isotype control (Adekar et al., 2008; Sharma et al., 2018b). The human mAbs were produced by the hybridoma cells adapted to 5% Ultra Low IgG fetal bovine serum (Life Technologies, Grand Island, NY) in Advanced RPMI 1640 (Thermo Fisher Scientific, Waltham, MA), and then incubated for 5 days in 500-ml roller bottles. Filtered supernatants were purified over protein G-Sepharose (Life Technologies). Antibody concentrations were determined using the NanoDrop spectrophotometer (Thermo Fisher Scientific). Antibodies were dissolved in phosphate buffered saline (PBS) and administered in two different concentrations: 0.02 μg/μl (i.e., 1X) and 0.2 μg/μl (e.g., 10X).

### Implantation of EEG Mount, Recording Electrodes, and Subcutaneous Micro-Osmotic Pumps

Mice were anesthetized with isoflurane and implanted with a unilateral injector guide cannula into the lateral ventricle, a 2 EEG/1 EMG head mount (Pinnacle Technology Inc., Lawrence, KS), and two subdural screw EEG electrodes to derive signals from the parietal and frontal cortex as previously described (Taraschenko et al., 2019, 2021). Mice were allowed to recover for 7 days in the recording chambers and then implanted with subcutaneous micro-osmotic infusion pumps (Alzet, Cupertino, CA) containing patient-derived monoclonal anti-NMDAR IgG or control 6A IgG. Mice were returned to the recording chambers, and their head mounts were attached to pre-amplifier and the EEG acquisition system (Pinnacle Technology Inc.). IgG solution was continuously perfused at a flow rate of 0.25 μl/h and EEG was continued for 14 days. Upon completion of the experiments, the contents of the pumps were examined for residual IgG solution.

### EEG Acquisition and Analysis

EEG signals recorded from the subdural screw electrodes were transmitted to a preamplifier unit connected by a tether to a conditioning/acquisition system. Signals were sampled at 400 Hz (preamplifier gain at 100X, total gain 5,000X, high pass EEG channel filter: 0.5 Hz, low pass EEG filter: 50 Hz), digitized using a 14-bit analog to digital converter and routed to a PC (Sirenia Seizure Pro 1.8.4., Pinnacle Technology Inc.). EEG and video analysis to identify electrographic and behavioral seizures were carried out retrospectively as previously described and verified visually without knowledge of the treatment status (Taraschenko et al., 2019, 2021). Seizures were defined as rhythmic EEG activity lasting 5 s or longer that exceeds the baseline amplitude by at least threefold; a modified Racine scale was applied to characterize the corresponding behavioral component of seizures as previously described by us (Taraschenko et al., 2019, 2021).

### Behavioral Tests

An open field test was performed to measure locomotor activity (total distance traveled) and anxiety-related behavior (percent time animals spent in the center of arena, 25% of the total area) using a video tracking system (Noldus, Leesburg, VA) as previously described (Taraschenko et al., 2019, 2021). Mice were habituated in the testing room for 30 min prior to being placed into the custom-made clear acrylic chamber (49 L × 49 W × 38 H cm) and were allowed to move freely during a 20-min trial. Total distance traveled and percent time spent in the center were calculated as previously described (Taraschenko et al., 2019, 2021). The intensity of illumination of the open field apparatus was 365 Lux.

### Preparation of Brain Tissue

Upon completion of experiments, animals were deeply anesthetized with isoflurane and transcardially perfused with ice-cold PBS solution. Brains were collected, and the two hemispheres were separated via sagittal cut along the midline. One hemisphere was immersed in 4% paraformaldehyde in PBS for 48–72 h; and the anterior one-fourth was separated, immersed in 30% sucrose solution for at least 24 h and flash frozen. The remaining part of the same hemisphere was transferred to 70% ethanol solution and stored at 4°C until it was paraffin-embedded for histology studies. The contralateral hemisphere was dissected into the hippocampus and other brain areas, flash frozen, and stored at −80°C for further studies.

### Immunohistochemistry

Paraffin-embedded coronal sections (9 μm; between bregma −1.55 and −2.03; at least 3 sections per mice) were processed for immunohistochemistry for GFAP (astrocytes) and Iba-1 (microglia). Briefly, sections were blocked with 2.5% horse serum for 30 min and were incubated at 4°C overnight with polyclonal rabbit anti-GFAP antibodies (1:500, N 1506, Dako, Carpentaria, CA) or anti-Iba-1 antibodies (1:500, 019-19741, Fujifilm Wako Chemicals, Richmond, VA). Following extensive washing, horse anti-rabbit secondary antibodies (Vectorlabs, Burlingame, CA) were used for signal detection.

### Imaging Acquisition and Processing

Slide specimens of the CA1 region of hippocampus were scanned with a Nuance Multi-Spectral Imaging System (Cambridge Research Instruments, Woburn, MA) fitted to a Nikon ECLIPSE 55i microscope (Nikon, Tokyo, Japan) with a 20X objective (1,392 × 1,040 pixels, 0.498 μm^2^/pixel). The absorbance profiles of immunostaining for GFAP or Iba-1 were determined as previously described (Taraschenko et al., 2021) and quantitative grayscale images corresponding to these profiles were extracted from the scanned images. Grayscale images were transferred to the Nuance environment for quantification of the intensities (gray scale units, gsu) and areas (μm^2^) as previously described (Taraschenko et al., 2021). The abundancies per event were computed as the product of mean pixel intensity and area (gsu • μm^2^). Immunopositivity for GFAP or Iba-1 in the hippocampal CA1 region of the antibody- and vehicle-treated mice was determined as previously described (Taraschenko et al., 2021).

### RNA Isolation and Quantitative Real-Time Polymerase Chain Reaction (qRT-PCR)

Total mouse hippocampus RNA was isolated using TRIzol (Invitrogen, Waltham, MA) with the PureLink RNA Mini Kit (Invitrogen). RNA concentration and purity were measured by A260 value and A260/A280 ratio, respectively. For cDNA synthesis 1 μg of total RNA, random primers, 4 μl qScript cDNA Supermix (Bio-Rad Laboratories, Hercules, CA) were used in a reaction volume of 20 μl at 25°C for 5 min, 42°C for 30 min, and 85°C for 5 min. Quantitative real-time PCR (qRT-PCR) was performed on mixtures of 2 μl 5X diluted cDNA, 1 μl 10 μM forward and reverse primers, and 10 μl iQ SYBR Green Supermix (Bio-Rad Laboratories) with a final volume of 20 μl in the iQ5 Multicolor Real-Time PCR Detection System (Bio-Rad Laboratories). Each sample was run in technical duplicates. PCR cycling conditions were as follows: 95°C for 2 min followed by 40 cycles of 95°C for 15 s and then 60°C for 1 min. Melting-curve analysis was used to verify single-species PCR product. Fluorescent data were acquired at the 60°C step. The geometric mean of cycle thresholds for glyceraldehyde 3-phosphate dehydrogenase (GAPDH), β-actin and hypoxanthine phosphoribosyl transferase 1 (HPRT1) was used as an internal control for each sample. Primers used for qRT-PCR for cyclooxygenase-2 (COX-2), interleukine-6 (IL-6), chemokine C-C motif ligand 2 (CCL-2), tumor necrosis factor (TNF), and IL-1β as well as GAPDH; β-actin and HPRT1 are shown in Table 1.

**TABLE 1:** Primers employed for quantitative real-time polymerase chain reaction (qRT-PCR) and their corresponding sequences.

Genes	Forward primer (5′–3′)	Reverse primer (5′–3′)
GAPDH	TGTCCGTCGTGGATCTGAC	CCTGCTTCACCACCTTCTTG
β-actin	AAGGCCAACCGTGAAAAGAT	GTGGTACGACCAGAGGCATAC
HPRT1	GGAGCGGTAGCACCTCCT	CTGGTTCATCATCGCTAATCAC
COX-2	CTCCACCGCCACCACTAC	TGGATTGGAACAGCAAGGAT
IL-1β	TGAGCACCTTCTTTTCCTTCA	TTGTCTAATGGGAACGTCACAC
IL-6	TCTAATTCATATCTTCAACCAAGAGG	TGGTCCTTAGCCACTCCTTC
TNF	TCTTCTGTCTACTGAACTTCGG	AAGATGATCTGAGTGTGAGGG
CCL2	CATCCACGTGTTGGCTCA	GCTGCTGGTGATCCTCTTGTA

*GAPDH, glyceraldehyde 3-phosphate dehydrogenase; HPRT1, hypoxanthine phosphoribosyl transferase 1; COX-2, cyclooxygenase-2; IL (interleukin)-1β; TNF, tumor necrosis factor chemokine; CCL2, C-C motif ligand 2.*

### Statistical Analysis

Seizure burden, locomotor activity, and anxiety scores in each treatment group were expressed as median total seizure counts over 2-weeks of infusion, total distance traveled and percent time spent in the center, respectively and the groups were compared using the one-way analysis of variance (ANOVA) test followed by Dunnett’s multiple comparison tests (GraphPad Prizm 8.4, San Diego, CA). Subgroup analyses of responses in mice treated with 1X and 10X protein concentrations were carried out using two-way ANOVA followed by Tukey’s or Sidak’s multiple comparison tests. The median latencies to the first seizure in four treatment groups were compared using the Kruskal-Wallis test. The mean immunopositivity for GFAP and Iba-1 in the hippocampus expressed as product of mean pixel intensity and area were compared using one-way ANOVA. The median fold change of hippocampal mRNA in mice treated with 5F5, 2G6, and 3C11 antibodies from the corresponding metabolite levels in mice with no instrumentation were compared for all inflammatory mediators with relevant values in mice infused with control 6A IgG using Kruskal-Wallis test followed by Dunn’s multiple comparison tests. Subgroup analyses of mRNA at 1X or 10X concentrations were carried out by means the Kruskal-Wallis test with Dunn’s correction.

## Results

### Seizure Burden Was Comparable During the Infusion of Three Different Monoclonal Antibodies

**FIGURE 1:**
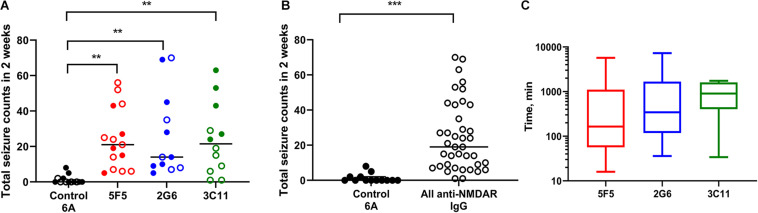
Administration of 5F5, 2G6, or 3C11 monoclonal anti-N-methyl-D-aspartate receptor (NMDAR) antibodies precipitated seizures in mice. **(A)** Seizure burden was similar in 5F5 (*n* = 15, red circles), 2G6 (*n* = 12, blue circles) and 3C11 (*n* = 12, green circles) antibody-treated groups. Data are median total seizure counts in 2 weeks. **p < 0.01, ANOVA with Dunnett’s multiple comparisons tests; open cycles, 1X antibodies; filled cycles, 10X antibodies. **(B)** The distribution of cumulative seizure counts in all anti-NMDAR antibody-treated groups. ***p < 0.001, *t*-test. **(C)** Time to the first seizure was not different between the four treatment groups (*p* = 0.39, Kruskal-Wallis test). Data are median latencies. Note log scale on Y axis.

**FIGURE 2:**
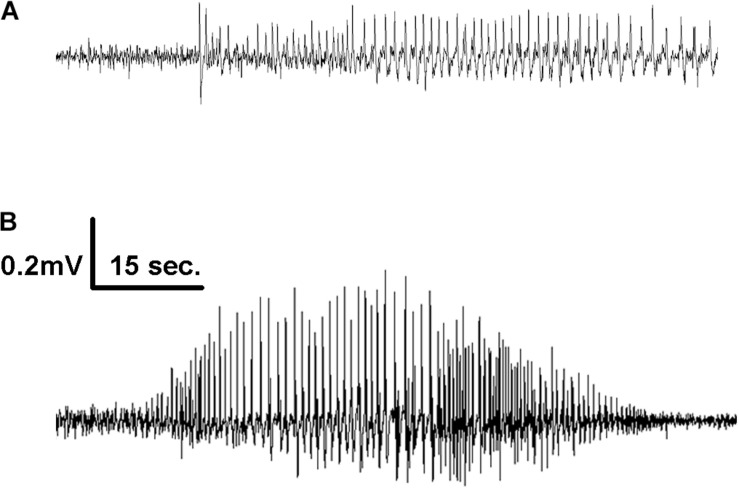
Representative EEG tracings of electrographic seizures recorded from the frontal cortex of mice infused with 1X **(A)** and 10X **(B)** 2G6 monoclonal anti-NMDAR antibodies.

All mice infused with 5F5 antibodies at two concentrations (*n* = 15) developed seizures. Similarly, 12 out of 14 mice (85.7%) infused with 2G6 antibody and 12 out of 13 mice infused with 3C11 antibodies (92.3%) exhibited seizures (Figure 1A). Mice that did not develop seizures were excluded from the analysis. During the 14-day infusion period, seizures occurred with median frequency of 21 (7–43; 25–75% interquartile range, IQR) per 2 week period, 14 (8.3–42.5), and 21.5 (6.8–39.5) in the 5F5, 2G6 or 3C11 antibody-treated mice, respectively over the entire period of infusion (Figures 1A,B). The seizure counts were significantly higher than in control 6A IgG-infused mice (*p* = 0.003, 5F5; *p* = 0.003, 2G6; *p* = 0.006, 3C11; Dunnett’s comparison tests). Consistent with our previous reports (Taraschenko et al., 2019, 2021), infrequent seizures also occurred in 4 out 18 (22.2%) control mice; and all 17 seizures were electrographic (Figures 2A,B). The median frequency of these seizures was 0 (0–2) per 2- week period.

The two antibody concentrations, 0.02 μg/μl (1X) and 0.2 μg/μl (10X), were selected such that the lowest concentration approximated the total CSF IgG protein level in encephalitis patients (Ly et al., 2018). At the higher dose (10X), all mice had seizures with 5F5 (*n* = 7) and 2G6 antibody (*n* = 7) exhibiting medians of 19 (6–43) and 14 (9–45) seizures, respectively, while those treated with 3C11 (*n* = 5) had a median of 43 (25.5–58) seizures (Figure 1A). At the lower dose (1X), 85% of all treated mice developed seizures. Specifically, 5F5 (*n* = 8), 2G6 (*n* = 5), and 3C11-infused mice (*n* = 7) developed seizures with median frequency of 22.5 (8.8–39.3), 14 (7.5–52.5), and 9 (1–19), respectively (Figure 1A). Only one out of five control mice infused with 1X 6A IgG developed seizures (0, 0–2). Three of 8 mice treated with 10X 6A IgG developed seizures with a median of 0 (0–4.3). While comparing the four treatment groups treated at two antibody doses, a statistically significant difference was found [*p* = 0.0007; *F*_(3, 44)_ = 6.86, two-way ANOVA]. Specifically, the mean number of seizures was significantly higher in the group treated with 10X 3C11 antibodies compared to the corresponding groups treated with 1X antibodies (*p* = 0.02, 1X vs. 10X, Sidak’s multiple comparison tests).

When combined into a single group, the antibody-treated mice demonstrated seizures with the cumulative median frequency of 19 (8–43) in 2 weeks (*p* = 0.0001, *t*-test, Figure 1B). The majority of seizures (96.9%) were electrographic (Figures 2A,B). The remaining few seizures (3%) were characterized by intermittent generalized myoclonic jerks or sustained clonic activity in the limbs (modified Racine’s scale 4 and 5, respectively). One seizure was characterized by behavioral arrest and three could not be visualized. The median time to the first seizure onset in 5F5, 2G6 or 3C11 antibody-treated groups were 164 (56.5–1100), 344 (118–1670), and 903 (403–1610) min, respectively (Figure 1C). The latency to the first seizure in the control group was 3040 (96.3–9741) min, but these differences did not reach statistical significance (*p* = 0.39, Kruskal-Wallis test).

### Locomotor Function in Mice Was Not Affected by the Administration of Different Anti-NMDAR Antibodies

**FIGURE 3:**
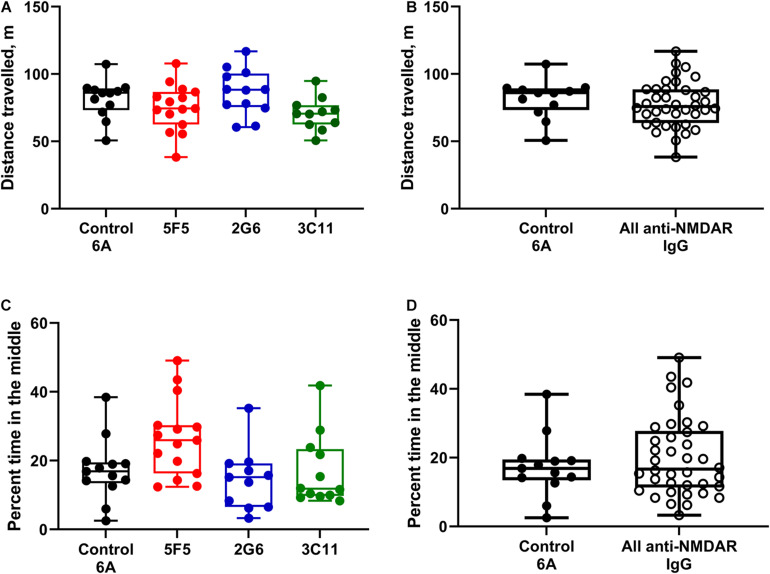
Administration of 5F5, 2G6, or 3C11 monoclonal anti-NMDAR antibodies did not affect mouse behavior. **(A)** Locomotor activity was similar in mice treated with different monoclonal antibodies and control 6A IgG (*p* = 0.08, ANOVA). *N* = 12 (control 6A IgG, black circles), *n* = 15 (5F5, red circles), *n* = 12 (2G6, blue circles), and *n* = 11 (3C11, green circles). **(B)** The distribution of total distance traveled in all anti-NMDAR antibody treated groups. **(C)** Despite the difference in the overall analysis, the anxiety scores in mice treated with either anti-NMDAR antibody were similar to those in control mice (*p* = 0.01, ANOVA; *p* = 0.1 vs. 5F5; *p* = 0.99 vs. 2G6; *p* = 0.99 vs. 3C11, Tukey tests). *N* = 13 (control 6A IgG, black circles), *n* = 15 (5F5, red circles), *n* = 11 (2G6, blue circles) and *n* = 12 (3C11, green circles). **(D)** The distribution of cumulative anxiety scores in anti-NMDAR antibody-treated groups. Data are medians (horizontal bars), 25–75% interquartile ranges (IQR, boxes), and the minimum and maximum values (whiskers, *p* = 0.43, *t*-test).

For the analysis of behavior, the groups of mice treated with 1X and 10X concentrations of antibody protein were first combined for the control 6A IgG-infused group and each monoclonal antibody group. The total distance traveled during a 20-min exploration of the open field (median ± IQR) in the control group (*n* = 12) was 86 (73.2–89.3) m. The corresponding distances in the 5F5 (*n* = 15), 2G6 (*n* = 12), and 3C11 (*n* = 11) antibody-treated groups were 74.5 (62.5–86.8), 88.3 (75.4–100.3), and 70.6 (62.6–76.9) m, respectively (Figure 3A). There was no significant difference between the four treatment groups (*p* = 0.08, ANOVA). Similarly, when the total distance traveled was compared among all the mice treated with monoclonal antibodies and control 6A IgG-treated animals, there was no significant difference (*p* = 0.43; *t*-test, Figure 3B).

When the subgroup analysis was performed using the two individual concentrations of antibody protein, the median distance traveled by mice infused with 1X (*n* = 5) and 10X (*n* = 7) control IgG protein were 86 (61.3–88.9) and 86.1 (77.1–90) m, respectively. The corresponding median values in the groups treated with 1X (*n* = 8) and 10X (*n* = 7) 5F5 antibodies were 77 (63.4–92.4) and 73.4 (56.6–83.1) m, respectively. The subgroup medians for 2G6 (*n* = 5, 1X and *n* = 7, 10X) and 3C11 (*n* = 6, 1X and *n* = 5, 10X) treated groups were 78.1 (76–96.7) and 88.6 (61.4–101) and 71.2 (60.6–81.4) and 70.6 (60.5–78) m, respectively. There were no differences in locomotor activity between the four groups of mice treated with two different antibody protein concentrations [*p* = 0.1, *F*_(3, 42)_ = 2.22, two-way ANOVA].

### Anxiety Scores Were Similar in Mice Treated With Anti-NMDAR and Control 6A IgG

Anxiety score expressed as percent time spent in the middle of arena in mice treated with 1X and 10X concentration of control 6A IgG (*n* = 13) was 16.9 (13.4–19.5). The percent time spent in the middle of the area was 25.9 (16.3–30.3) in mice treated with both concentrations of 5F5 anti-NMDAR antibodies (*n* = 15). The corresponding anxiety scores were 15.2 (6.5–19.2) and 11.8 (9.7–23.3) in mice treated with both concentrations of 2G6 (*n* = 11) and 3C11 antibody protein (*n* = 12), respectively (Figure 3C). The overall comparisons of medians revealed a significant effect of treatment on the anxiety scores in mice (*p* = 0.01; ANOVA); however, there were no differences between the control 6A antibody-infused and either of the antibody-treated groups (*p* = 0.1, vs. 5F5; *p* = 0.99, vs. 2G6; *p* = 0.99, vs. 3C11, Tukey tests). Further, there were no differences between the anxiety scores of mice in the combined anti-NMDAR antibody-treated group and control group (*p* = 0.42, *t*-test, Figure 3D).

When disaggregated with respect to two antibody concentrations, the median percent times spent in the middle in control mice infused with the 1X (*n* = 5) and 10X (*n* = 8) IgG protein were 19.0 (12.6–27.8) and 15 (2.5–38.4), respectively. The corresponding values in the groups treated with 5F5 antibodies were 25.4 (12.6–30.3) and 27.4 (12.4–49.1) percent, respectively (*n* = 8, 1X and *n* = 7, 10X). The median anxiety scores in mice treated with 1X (*n* = 4) and 10X (*n* = 7) of 2G6 antibodies were 18.1 (15.7–35.2) and 8.3 (3.3–19.6), respectively while the corresponding values for the 3C11 subgroups were 21.8 (10.4–41.8) and 9.6 (8.3–11.6) (*n* = 7, 1X and *n* = 5, 10X). The overall comparison of four treatment groups at two different concentrations revealed that the antibody concentration tended to affect the anxiety scores [*p* = 0.046, *F*_(3, 43)_ = 4.12; two-way ANOVA]; however, the *post hoc* comparisons did not reveal any significant differences between the groups treated with the corresponding 1X and 10X antibodies (*p* = 0.96, 6A; *p* = 0.8, 5F5; *p* = 0.21, 2G6; *p* = 0.11, 3C11, Sidak’s comparison tests).

### Expression of Astrocytic and Microglial Markers of Inflammation in the Hippocampus Were Not Affected by Antibody Treatment and Seizures

The immunopositivity for GFAP and Iba-1 in the hippocampus was assessed in the combined datasets of mice treated with 1X and 10X concentrations of anti-NMDAR and control 6A antibody proteins and the subgroup analysis for each concentration was omitted due to the small number of animals.

**FIGURE 4:**
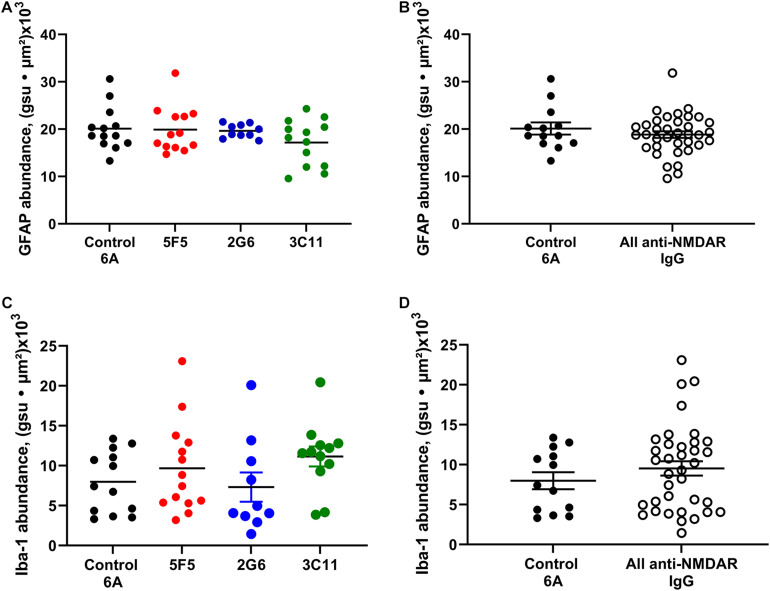
Expression of astrocytic and microglial markers of inflammation in the CA1 region of hippocampus of mice with anti-NMDAR antibody-induced seizures. The expression of GFAP in 1X and 10 × 5F5, 2G6, or 3C11 antibody-treated mice **(A)** and combined antibody-treated mice **(B)** was similar to that in control 6A IgG-infused mice. The expression of Iba1 in 5F5, 2G6 or 3C11- antibody treated mice **(C)** and combined antibody-treated mice **(D)** was comparable in all groups and similar to that in control 6A IgG-infused mice. GFAP: *n* = 13 (control 6A IgG, black circles), *n* = 13 (5F5, red circles), *n* = 10 (2G6, blue circles), and *n* = 13 (3C11, green circles). Iba1: *n* = 13 (control 6A IgG), *n* = 14 (5F5), *n* = 10 (2G6), and *n* = 12 (3C11). The abundance of GFAP or Iba1 labeling in the CA1 region was determined as the sum of the products of mean pixel intensity (gray scale units, gsu) and area of each event (μ^2^) in a fixed scan area. The data are mean ± SEM.

The expression of GFAP in the CA1 region of hippocampus of mice treated with 1x and 10X control 6A IgG protein (*n* = 13) was (mean ± SEM) 20.1 ± 1.3 gsu • μm^2^ × (10^3^). The expression of GFAP in 5F5, 2G6, and 3C11 groups combined for both concentrations were 19.9 ± 1.3 (*n* = 13), 19.6 ± 0.5 (*n* = 10) and 17.2 ± 1.3 (*n* = 13) gsu • μm^2^ × (10^3^), respectively (Figure 4A). There were no significant differences among the four treatment groups (*p* = 0.41, ANOVA). When all anti-NMDAR-antibody-treated groups were combined (*n* = 36) there was no significant difference in GFAP expression between the antibody-infused and control 6A antibody-infused mice (*p* = 0.37, *t*-test, Figure 4B).

The expression of Iba-1 in the CA1 region of hippocampus in mice treated with 1X and 10X antibody protein (*n* = 13) was 8.0 ± 1.1 (mean ± SEM) gsu • μm^2^ × (10^3^). The expression of Iba-1 in 5F5, 2G6, and 3C11 groups was 9.7 ± 1.5 (*n* = 14), 9.7 ± 1.5 (*n* = 10), and 11.2 ± 1.3 (*n* = 12) gsu • μm^2^ × (10^3^), respectively (Figure 4C). The ANOVA did not reveal any significant difference between the four treatment groups (*p* = 0.41). Further, there was no significant difference between the combined anti-NMDAR antibody-treated groups (*n* = 36) and control 6A antibody-infused mice (*p* = 0.35, *t*-test, Figure 4D).

### Analysis of Cytokines in the Hippocampus of Mice With Seizures

**FIGURE 5:**
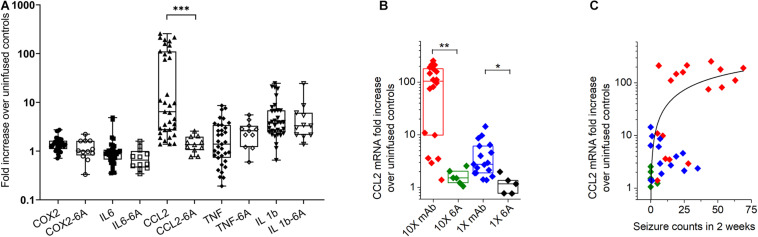
Expression of inflammatory mediators in the hippocampus of mice with antibody-induced seizures. **(A)** C-C chemokine ligand 2 (CCL2) mRNA level was increased in mice that developed seizures during the infusion of antibodies. Data are median fold change in mRNA expression (horizontal bars) in mice treated with 1X and 10X concentrations of 5F5, 2G6 and 3C11 antibody protein (closed symbols) or 6A control IgG protein (open symbols), relative to the corresponding values in mice that were not infused with antibodies. *N* = 34–36 (5F5, 2G6 and 3C11 antibodies), *n* = 11–12 (6A antibodies). The boxes are interquartile ranges (IQR, 25–75%), the whiskers are the minimum and maximum values. ***p < 0.001, Kruskal-Wallis test with Dunn’s multiple comparison tests. COX-2, cyclooxygenase-2; IL-6, interleukin-6 (IL-6); TNF, tumor necrosis factor **(B)** The increase of hippocampal CCL2 mRNA was related to 5F5, 2G6, and 3C11 monoclonal antibody protein (mAb) concentration. Data are medians (horizontal bars), 25–75% interquartile ranges (IQR, boxes). Note log scale on Y axis. **(C)** Increase in CCL2 mRNA expression in hippocampus of mice treated with 10 × 5F5, 2G6 and 3C11 monoclonal antibody protein was related to the number of antibody-induced seizures. 1x: *n* = 14 (mAb, blue diamonds) and *n* = 5 (control 6A IgG, black diamonds); 10X: *n* = 18 (mAB, red diamonds) and *n* = 6 (control 6A IgG, green diamonds). Note log scale on Y axis. **p < 0.01, and *p < 0.05, Kruskal-Wallis test with Dunn’s correction.

The effect of antibody-induced seizures on mRNA expression in the hippocampus of mice was examined for five different inflammation-associated genes on day 17 following the initiating of antibody infusion, which was approximately 3 days after infusion had stopped. The mRNA levels of COX-2, IL-6, CCL2, TNF, and IL-1β were normalized to their respective means of 7 mice that had not been infused with either antibody or IgG. Data from mice infused with 1X and 10X concentrations of 5F5, 2G6, and 3C11 protein were combined (Figure 5A). The fold change (median ± IQR) in the mRNA expression in mice over the corresponding values in untreated mice for COX-2 were 1.39 (1.1–1.6) in the combined antibody treated group (*n* = 36) and 1 (0.8–1.6) in 6A-treated group (*n* = 12). The corresponding values in antibody-treated and 6A-infused mice for IL-6 (*n* = 36), TNF (*n* = 35), and IL-1β (*n* = 34) were 0.9 (0.6–1.1) and 0.7 (0.5–1), 1.4 (0.7–3.4) and 2.6 (1.2–3.3), 3.7 (2.4–6.9), and 2.6 (1.2–3.3), respectively (*n* = 11–12, 6A IgG-infused control group). The CCL2 mRNA levels were 6.4 (2.7–110) in the monoclonal antibody-treated group (*n* = 35) and 1.3 (1.1–2.0) in the control 6A IgG-infused group (*n* = 11). The overall Kruskal-Wallis test showed a significant difference between mRNA fold changes between 10 groups (*p* = 0.0001; 10 groups with 233 total values; Kruskal-Wallis statistic = 121.4). When comparing antibody-infused mice with mice infused with 6A control IgG antibodies, CCL2 was the only mediator that showed a significant, 4.8 times increase in mRNA expression (*p* = 0.0003, Dunn’s test, Figure 5A).

When the CCL2 mRNA levels were disaggregated in respect to antibody concentration, groups treated with 5F5, 2G6, or 3C11 proteins at 10X and 1X concentrations had significantly elevated CCL2 mRNA levels compared to the corresponding 6A IgG-infused control groups (*p* = 0.0045 and *p* = 0.046, Kruskal-Wallis test with Dunn’s corrections; Figure 5B). The CCL2 mRNA expression level in mice treated with 1X and 10X of 5F5, 2G6, or 3C11 monoclonal antibodies was related to seizure burden as measured with total seizure counts in 2 weeks (Figure 5C). The R^2^ value for the fit to the Hill equation between the seizure number and CCL2 mRNA levels was 0.35.

## Discussion

We found that three distinct monoclonal anti-NMDAR antibodies from a single encephalitis patient with seizures that bind at partially overlapping epitopes within the N-terminal domain (NTD) of the GluN1 subunit induce seizures in mice; the seizures were of comparable severity. The monoclonal antibodies did not have an effect on motor or anxiety behaviors in mice. While both antibody administration and seizures did not alter the expression of astrocytic and microglial markers of inflammation in the hippocampus, mice treated with antibodies demonstrated an increased mRNA expression of hippocampal CCL2, a pro-inflammatory chemokine known to be relevant for the persistence of seizures in other seizure models (Foresti et al., 2009; Tian et al., 2017; Varvel et al., 2016, 2021). Higher CCL2 expression correlated with higher seizure burden. Neuropsychiatric, cognitive, motor, and seizure phenotypes of patients with anti-NMDAR encephalitis vary significantly; and it is not clear whether or how the unique repertoires of antibodies contribute to such variable presentations (Dalmau et al., 2008; Kolls et al., 2020). It is expected that physiological effects of a polyclonal antibody response on the brain are defined by a particular combination of the existing functional antibodies (Diamond et al., 2009). The pathogenic potential of antibodies in autoimmune encephalitis is supported by resolution of clinical symptoms following the removal of antibodies with plasmapheresis or after B-cell depleting therapies (Dalmau et al., 2008, 2011). In the present study, we reproduced previously reported pathogenic effects of anti-NMDAR antibodies on seizures in rodents (Wright et al., 2015; Taraschenko et al., 2019, 2021). In addition, we found that two other monoclonal antibodies derived from the same patient with seizures but targeting different epitopes within the NTD precipitated seizures in mice and these seizures were similar in intensity to the other monoclonal antibody-treated group.

Published work on anti-NMDAR antibody epitopes is scarce (Gleichman et al., 2012; Ogawa et al., 2016) and is largely focused on IgG from encephalitis patients that recognize the NTD-G7 domain (N368/G369) (Gleichman et al., 2012). Other case series in anti-NMDAR encephalitis patients and patients with acute neuropsychiatric manifestations of systemic lupus erythematosus who tested positive for anti-NMDAR IgG found that patients’ antibodies recognized an epitope in the NTD outside of G7 domain (Ogawa et al., 2016; Castillo-Gomez et al., 2017) although anti-GluN2A/B antibodies detected in patients with lupus do not alter synaptic transmission permanently but rather induce neuronal death (Husebye et al., 2005; Tay and Mak, 2015). Taken collectively, these findings suggest that antibody-mediated ion channel dysfunction and the *in vivo* effects of the antibodies depend on the epitope targeted, and neuropsychiatric manifestations associated with anti-NMDAR IgG positivity are connected with NTD or NTD-G7 epitopes (Joubert and Honnorat, 2015; Ogawa et al., 2016). To our knowledge, no such studies have been published on the relevance of encephalitis antibody epitopes to manifestation of seizures or anxiety. Our findings support the premise that the epitope location may not be critical for the manifestation of seizures in anti-NMDAR encephalitis and other factors may contribute to the diversity of clinical phenotypes in these patients.

The severity of clinical phenotype of anti-NMDAR encephalitis, including the neuropsychiatric manifestations of the disease, rely on the circulating antibody titers (Dalmau et al., 2008). Specifically, poor outcomes in patients with anti-NMDAR encephalitis are correlated with increased serum and CSF antibody titers and relapses are linked to the increase in CSF antibody titers (Dalmau et al., 2011; Gresa-Arribas et al., 2014; Joubert and Honnorat, 2015). However, while such a relationship between the antibody titer and disease severity was demonstrated within the same patients, a titer-phenotype correlation among a population of patients was not found. That leads to the premise that in polyclonal responses, the clinical picture may be influenced by antibody titer, the epitopes targeted and their distinct biophysical properties, such as antibody affinities (Ly et al., 2018). Moreover, it was proposed that while low affinity antibody in patient specimens may not be detected in conventional cell-based assays, these antibodies may still contribute to the clinical phenotype (Ly et al., 2018). In our model, the administration of monoclonal antibodies at the high concentration did not result in more frequent seizures than the low concentration; therefore, the antibody titers may not be the key determining factors of seizure burden. Thus, the concentrations of antibody protein chosen for our study were in the range of antibody IgG estimated to be present in CSF of encephalitis patients (i.e., 0.02–0.05 μg/μl), although the CSF signal in the latter report was predominantly represented by higher-affinity antibodies (Ly et al., 2018). Therefore, it is possible that the highest protein concentration chosen for our study was not high enough to significantly increase seizure burden in mice, or that antibody affinities were low. Further, it is possible that the individual biological responses elicited by each monoclonal antibody may need to be executed in a concerted fashion to produce measurable changes in behavioral responses. Consistent with this premise, the frequency of antibody-induced seizures in the present study was lower than that in our previous studies where we infused patient CSF or purified patient IgG fraction that harbor polyclonal anti-NMDAR antibodies (Taraschenko et al., 2019, 2021). Thus, CSF or a combination of multiple distinct antibodies could be more effective to induce seizure responses than any single monoclonal antibodies. Future studies using combinations of monoclonal antibodies may allow to clarify this theory.

We found that anxiety scores in mice treated with any of the three monoclonal antibodies did not differ from those in control mice. This is in contrast to the findings of anxiety-like behavior reported in studies using other mouse models of anti-NMDAR encephalitis (Jones et al., 2019). However, the active immunization with NMDA holoreceptors used by Jones et al. (2019) was different from passive transfer of anti-NMDAR antibodies used in our model. The former method led to the development of florid encephalitis in mice and their behavioral assessments were made much later (i.e., at 6 weeks post-treatment) compared to our timing (i.e., at 2 weeks from the initial exposure to antibodies). Further studies with more robust assessment of this behavioral domain following the exposure to various monoclonal antibodies or treatment with antibody combinations will clarify the biological significance of these findings.

Syndromic manifestations of anti-NMDAR encephalitis are largely attributed to the intrathecally produced anti-NMDAR antibodies (Tuzun et al., 2009; Dalmau, 2016). However, serum autoantibodies generated in the secondary lymphoid organs can also contribute to the expression of disease phenotype if they gain access to the CNS through a compromised blood–brain –barrier (BBB) (Diamond et al., 2009; Castillo-Gomez et al., 2016). Increased BBB permeability can be triggered by systemic inflammation caused by a preceding exposure to viral pathogens or be induced by repeated seizures (Marchi et al., 2012; Pruss et al., 2012; Armangue et al., 2014; Omae et al., 2018; Swissa et al., 2019). While it is not entirely clear if antibodies are being transported by transcellular endocytosis or paracellular route, several pro-inflammatory mediators, including CCL2, are critically involved in the migration of the antibodies to the brain (Banks, 2005; Banks and Erickson, 2010; Wesselingh et al., 2019). We found that CCL2 mRNA expression is increased in the hippocampus of mice with recurrent seizures induced by monoclonal antibodies. Moreover, the higher seizure burden in mice treated with anti-NMDAR antibodies was associated with higher expression levels of this chemokine. Other studies in patients with intractable temporal lobe epilepsy and rodents with induced seizures have demonstrated the upregulation of CCL2 in the hippocampus and other brain areas (Wu et al., 2008; Banks and Erickson, 2010; Varvel et al., 2016; Orsini et al., 2021). In pharmacoresistant epilepsy, CCR2 is highly expressed in neurons and monocytes suggesting that CCL2 and its receptor may play a role in epileptogenesis (Bozzi and Caleo, 2016; Varvel et al., 2016). CCR2 is related to the regulation of other pro-inflammatory mediators and was shown to be required for production of IL-1β after convulsant-induced status epilepticus (Varvel et al., 2016; Tian et al., 2017). We did not detect a significant change in the expression of IL-1β or other cytokines on day 17 following the start of antibody infusion. One potential reason could be that expression of mRNA for these cytokines in seizures peaks and falls at faster rates than CCL2 (Jiang et al., 2015; Liba et al., 2016). Thus, in patients with anti-NMDAR encephalitis triggered by herpes simplex virus infection, the initial surge of proinflammatory mediators in CSF, including IL-1β, TNF and CCL2 during the prodromal phase was followed by a sustained elevation of CCL2 but a decrease of other cytokine concentrations at the onset of clinical symptoms in 2 weeks (Omae et al., 2018). Consistent with this, in our previous study we demonstrated the reduction of seizures and attenuation of hippocampal inflammation by a systemic administration of selective IL-1 receptor antagonist, anakinra, on days 6–11 in a similar antibody infusion paradigm (Taraschenko et al., 2021), raising the possibility that translational regulation of IL-1β via the inflammasome might be more relevant to anakinra action than transcriptional upregulation. Another potential reason for not finding the increased expression of other chemokines and cytokines is that seizures caused by individual monoclonal antibodies are not intense or frequent enough to cause a strong inflammatory reaction. Consistent with this premise, we did not find overt microgliosis or astrogliosis in this study. This is in contrast to our previous studies where we demonstrated higher seizure burden and a broad inflammatory response in the hippocampus during infusion of patient CSF or purified IgG that contained a full repertoire of anti-NMDAR antibodies (Taraschenko et al., 2019, 2021). The lack of increased expression of astrocytes and microglia in the present study might be explained by insufficient seizure severity in mice infused with monoclonal antibodies compared to those exposed to the patient CSF or purified patient IgG in our earlier studies. The lower seizure frequency could be because the concentration of the monoclonal antibodies used in this study was lower than that in the CSF of the patient from whom the antibodies were derived. Alternatively, the monoclonal antibodies need to be delivered in combination in order to produce seizure and inflammatory phenotype similar to that observed in mice treated with patient CSF or purified patient IgG (Taraschenko et al., 2019, 2021). The effects of combined monoclonal antibodies will be assessed in the future studies.

Despite the thorough characterization of cellular effects of anti-NMDAR antibodies, many experimental and clinical questions regarding antibody pathogenicity and effects on the brain networks remain unanswered. The development of monoclonal anti-NMDAR antibodies obviates the need to rely on CSF supply from affected patients and provides a powerful tool to study the biological effects of antibodies in encephalitis models. In this study, we found that monoclonal antibodies with different binding epitopes derived from the same patient resulted in a similar seizure phenotype. Passive transfer of monoclonal antibodies and seizures results in an increased CCL2 mRNA expression in the hippocampus without overt alteration of astrogliosis and microgliosis.

## Data Availability Statement

The raw data supporting the conclusions of this article will be made available by the authors, without undue reservation.

## Ethics Statement

The animal study was reviewed and approved by the Institutional Animal Care and Use Committee (IACUC) of the University of Nebraska Medical Center (UNMC).

## Author Contributions

All authors listed have made a substantial, direct and intellectual contribution to the work, and approved it for publication.

## Conflict of Interest

The authors declare that the research was conducted in the absence of any commercial or financial relationships that could be construed as a potential conflict of interest.

## Publisher’s Note

All claims expressed in this article are solely those of the authors and do not necessarily represent those of their affiliated organizations, or those of the publisher, the editors and the reviewers. Any product that may be evaluated in this article, or claim that may be made by its manufacturer, is not guaranteed or endorsed by the publisher.
